# Cross-sectional and longitudinal quantification of total white matter perivascular space volume fraction in Dutch-type Cerebral Amyloid Angiopathy

**DOI:** 10.1016/j.nicl.2025.103778

**Published:** 2025-04-05

**Authors:** Manon R. Schipper, Thijs W. van Harten, Arie-Tjerk Razoux-Schultz, Kanishk Kaushik, Lydiane Hirschler, Sabine Voigt, Ingeborg Rasing, Emma A. Koemans, Rosemarie van Dort, Reinier G.J. van der Zwet, Sanne E. Schriemer, Erik W. van Zwet, Jeroen van der Grond, Mark A. van Buchem, Steven M. Greenberg, Marieke J.H. Wermer, Matthias J.P. van Osch, Marianne A.A. van Walderveen, Sanneke van Rooden

**Affiliations:** a Department of Radiology, Leiden University Medical Center, Leiden, the Netherlands; b Department of Neurology, Leiden University Medical Center, Leiden, the Netherlands; c Department of Biostatistics, Leiden University Medical Center, Leiden, the Netherlands; d Hemorrhagic Stroke Research Program, J Philip Kistler Research Center, Department of Neurology, Massachusetts General Hospital, Boston, MA, USA; e Department of Neurology, University Medical Center Groningen, Groningen, the Netherlands

**Keywords:** Perivascular spaces, Brain clearance, Amyloid-β, Cerebral amyloid angiopathy, Dutch-type cerebral amyloid angiopathy, Hereditary cerebral hemorrhage with amyloidosis Dutch-type, Small vessel disease

## Abstract

Enlarged perivascular spaces (PVS) in the centrum semiovale are an important marker of Cerebral Amyloid Angiopathy (CAA) and are thought to reflect brain clearance dysfunction. However, the current golden standard for assessing PVS is limited to a unilateral, single slice, qualitative analysis, which has the disadvantage of a strong ceiling effect. We aim to introduce a whole-brain PVS volume fraction (PVS_vf_) measurement to assess cross-sectional and longitudinal PVS_vf_ differences between pre-symptomatic and symptomatic Dutch-type CAA (D-CAA) mutation carriers and similar-age controls. PVS_vf_ was assessed with a Frangi-vesselness filter-based, segmentation tool developed in-house and was compared cross-sectionally in 70 participants (28 symptomatic D-CAA, 17 pre-symptomatic D-CAA, 10 controls > 50 years, 17 controls ≤ 50 years) and longitudinally in 40 participants (16 symptomatic D-CAA, 13 pre-symptomatic D-CAA, 11 controls combined from both age groups). We found a higher baseline PVS_vf_ in symptomatic D-CAA compared to controls ≤ 50 years (*p* < 0.0001, 95% CI [−0.051, −0.025]) and controls > 50 years (*p* < 0.0001, 95% CI [-0.042, −0.016]), in pre-symptomatic D-CAA compared to controls ≤ 50 years (*p* = 0.023, 95% CI [−0.035, −0.002]), and in controls > 50 years compared to controls ≤ 50 years (*p* < 0.001, 95% CI [0.004, 0.014]). We found no group differences in PVS_vf_ change over time. The introduction of this quantitative measure of PVS volume in D-CAA showed cross-sectional differences already in pre-symptomatic D-CAA, indicating increased PVS_vf_ in the early stages of D-CAA. We did not observe longitudinal differences over a four-year follow-up when analyzed at group level.

## Highlights

• Novel whole-brain semi-automated perivascular space segmentation-method in D-CAA.
• Cross-sectional and longitudinal perivascular space volume fraction (PVS_vf_) in D-CAA.
• Increased PVS_vf_ in early stages of Dutch-type Cerebral Amyloid Angiopathy (D-CAA)
• No difference in PVS_vf_-change over 4 yr follow-up between D-CAA and control subjects.

## 1 Introduction

Cerebral Amyloid Angiopathy (CAA) is a cerebral small vessel disease that is characterized by cerebrovascular amyloid-β accumulation, specifically in the walls of the leptomeningeal and cortical vessels (Vonsattel et al., 1991). CAA is one of the leading etiologies of lobar intracerebral hemorrhage (ICH) in the elderly (Aguilar and Brott, 2011). Dutch-type CAA (D-CAA) is an autosomal dominant hereditary form of CAA that is caused by a point mutation in the amyloid protein precursor gene (Maat-Schieman et al., 2005). D-CAA is biologically, chemically, and clinically very similar to sporadic CAA, making it an ideal model for studying CAA. However, D-CAA has an earlier onset and presents with a more progressive disease course than sporadic CAA. Genetic testing enables an early, definite diagnosis that allows to study the disease in the pre-symptomatic stage as well as at a younger age when comorbidities are still rare.

The current pathophysiological model for CAA is that amyloid-β accumulation causes vessel rigidness, limits smooth muscle cell functioning, and promotes vessel wall damage which makes these vessels prone to rupture and impairs cerebral hemodynamics (Koemans et al., 2023). It is hypothesized that the accumulation of vascular amyloid-β in CAA is related to impaired amyloid-β clearance (van Veluw et al., 2024). Perivascular spaces (PVS) – cerebrospinal fluid (CSF) filled spaces that surround cerebral vessels, mainly arteries and arterioles (Mestre et al., 2017) – are thought to be an important pathway in clearing waste products from the brain to the veins and lymphatic vessels (Rasmussen et al., 2022). Previous research, looking at both in-vivo and ex-vivo MRI and histopathology, has shown that enlarged MRI visible PVS in the centrum semiovale white matter are linked to vessels affected by amyloid-β accumulation in the overlying cortex (Perosa et al., 2022). The underlying pathological process might be that amyloid-β related vessel rigidness in the cortical region of the vessel limits vasoreactivity, which is thought to be one of the main drivers of CSF motion (Van Veluw et al., 2020). In turn, this may block PVS CSF flow towards the subarachnoid space, thereby inflating its volume (Brown et al., 2018).

While PVS seem to appear in every cerebral lobe, MRI visible enlarged PVS in CAA have been mostly studied in the centrum semiovale – CSO-EPVS – and are found to be increased in sporadic CAA as well as in the symptomatic phase of D-CAA (Martinez-Ramirez et al., 2018). To our knowledge, the only longitudinal assessment of PVS in CAA thus far has been done in the EDAN (Early Diagnosis of Amyloid Angiopathy Network) study (Van Dijk et al., 2022). This study showed a slight but non-significant increase in PVS based on a visual rating scale in controls and no progression in pre-symptomatic and symptomatic D-CAA mutation carriers over a four-year follow-up (Van Dijk et al., 2022, Potter et al., 2015). The visual rating scale considers the PVS count on a single unilateral slice in the CSO and categorizes this count on a scale from 0 to 4 (0: no EPVS, 1: ≤ 10 EPVS, 2: 11 – 20 EPVS, 3: 21 – 40 EPVS, and 4: > 40 EPVS) (Potter et al., 2015). In symptomatic D-CAA participants; the detection of progression was impossible, as all participants were already classified into the fourth and, thus, highest category at the baseline measurement. As this strong ceiling effect of the visual rating scale limits the sensitivity of measuring disease progression, the authors proposed that future studies should apply a (semi-)quantitative volume measurement to assess progression of PVS (Van Dijk et al., 2022). The continuous scale of volumetric measurements will circumvent the ceiling effect that was introduced through the visual rating scale. In addition, a (semi-)quantitative measure would enable a whole-brain assessment of PVS, allowing a more complete consideration of PVS and offering robustness even with regional differences.

Currently available neural network algorithms to quantify PVS have been trained in healthy individuals, normal aging, mild cognitively impairment, Alzheimer’s disease, and atherosclerosis (Lan et al., 2023, Choi et al., 2020, Lian et al., 2018, Huang et al., 2024, Rashid et al., 2023) and other vesselness filter-based pipelines have been tested and developed in community dwelling individuals, healthy individuals, Parkinson’s disease (Sepehrband et al., 2019, Donahue et al., 2022, Ballerini et al., 1936). We expect that methods developed for and trained on non-CAA populations will fall short in dealing with gross pathology, in the PVS region of interest (ROI), that is often involved in CAA – e.g. ICH (clusters) and extensive white matter hyperintensities (WMH) (Fandler-Hofler et al., 2024). Thus far, one study has assessed PVS volume in (D-)CAA, however, this quantification was limited to a single slice and a cross-sectional study design (Martinez-Ramirez et al., 2018).

Considering the limitations of the current gold standard for PVS assessment and unavailability of other PVS assessment methods for CAA, we first aim to develop and assess a method to quantify PVS volume throughout the entire normal-appearing white matter (NAWM) of the cerebrum in pre-symptomatic and symptomatic D-CAA mutation carriers in comparison to controls. Based on previous research we hypothesize to find higher PVS volume fraction in symptomatic D-CAA compared to controls similar in age. Second, we aim to study group differences in normalized PVS volume at baseline and its progression after four-years follow-up. We hypothesize to find increased progression in symptomatic D-CAA compared to controls similar in age. Performance of the PVS volume segmentations will be inspected in comparison to the visual rating scale scores.

## 2 Methods

### 2.1 Study design and participants

Patients and controls were retrieved from the aforementioned EDAN study (Van Dijk et al., 2022) and the prospective natural history study on D-CAA (the AURORA study). The EDAN study was a collaboration between the Leiden University Medical Center (LUMC), Massachusetts General Hospital, and the Erasmus University Medical Center. The study included a baseline visit (between 2013 and 2014) and a follow-up visit after approximately four years. The AURORA study was performed at the LUMC, for the current study data from the baseline visit (between 2018 and 2020) and follow-up visit after approximately three years was included. Participants were recruited via the LUMC (outpatient) clinic. For both studies, the inclusion criteria were age ≥ 18 years and no contraindications for 3.0 Tesla MRI. To be eligible for the current study, AURORA participants needed to have a 2D T2-weighted MRI scan at baseline and follow-up after three years, i.e. similar to the EDAN protocol.

All participants underwent genetic testing for the Glu693Gln point mutation in the APP gene. Participants were diagnosed by a DNA proven APP mutation or a medical history of ≥ 1 lobar ICH and ≥ 1 first-degree relative with D-CAA. Participants were considered as control participants in absence of the D-CAA mutation, and additional control subject were recruited in the EDAN study. Participants who did not want to know their mutation status were not informed about the genetic testing results. The controls were divided into two groups to be similar in age and sex to the pre-symptomatic and symptomatic D-CAA mutation carriers. For the current study, symptomatic D-CAA mutation carriers were defined as mutation carriers who had experienced at least one symptomatic ICH that was confirmed on CT or MRI. Since body-mass index (BMI) has previously been shown to be related to PVS volume (Barisano et al., 2021), BMI was calculated retrospectively based on self-reported weight and height.

Both studies were approved by the Medical Ethics Committee Leiden-The Hague-Delft and written informed consent was obtained from all participants before enrollment.

### 2.2 MRI data acquisition

MRI scans of all participants were acquired on a whole-body 3.0 Tesla magnetic resonance system (Philips Achieva, Best, the Netherlands) with a standard 32-channel head coil. Scans that were used in the current study are: three-dimensional T1-weighted images (repetition time (TR)/echo time (TE) = 9/4.6 ms, flip angle (FA) = 8°, 140 slices, and field of view (FOV) = 224 x 178.5 x 168 mm with a voxel size of 1.17 x 1.17 x 1.20 mm, resulting in a scan duration of ∼ 5 min); multi-slice T2-weighted images (TR/TE = 4200/80 ms, FA = 90°, 40 slices with no interslice gap, and FOV = 224 x 180 x 144 mm with a voxel size of 0.50 x 0.56 x 3.60 mm, resulting in a scan duration of ∼ 3 min); three-dimensional fluid-attenuated inversion recovery images (FLAIR; TR/TE = 4800/266 ms, inversion time = 1650 ms, 321 slices, and FOV = 250 x 250 x 180 mm with a voxel size of 1.11 x 1.11 x 1.12 mm, resulting in a scan duration of ∼ 5 min); susceptibility-weighted images (TR/TE = 45/31 ms, FA = 13°, 140 slices with no interslice gap, and FOV = 250 x 175 x 112 mm with a voxel size of 0.78 x 0.78 x 1.60, resulting in a scan duration of ∼ 6 min). A fraction of the T2-weighted scans – the scans from the AURORA protocol – had slightly different parameters, namely a 3.0 mm slice thickness and a TR of 4744 ms. Full description of the relevant scans from the AURORA 3.0 Tesla MRI protocol can be found in Supplementary Table S1.

### 2.3 Quantification of perivascular spaces

#### 2.3.1 Region of interest formation

First, white matter ROIs for PVS assessment were created for each participant, through the following steps: 1) registration of the FLAIR image to the T1-weighted image (Penny and Ashburner, 2006); 2) multimodal segmentation, based on the T1 and the registered FLAIR image (Penny and Ashburner, 2006); 3) brain extraction of the T1-weighted image using optiBET (Lutkenhoff et al., 2014); 4) 2.5 mm dilation of the CSF segmentation resulting from the multimodal segmentation (from step two); 5) removal of the dilated CSF segmentation from the brain extracted T1 mask; 6) registration of cerebellum (Diedrichsen et al., 2009), brainstem, basal ganglia, and lateral ventricles templates (Desikan et al., 2006) to the T1-weighted image, using FSL’s linear registration (Jenkinson MaS, 2001, Jenkinson et al., 2002); 7) removal of the templates from step six from the ROI. This resulted in an ROI restricted to the white matter of the cerebrum (Fig. 2.1).

#### 2.3.2 PVS volume fraction (PVS_vf_) assessment

Total PVS volume was quantified using in-house developed software in MeVisLab 3.4.2 (Mevislab.de. MeVisLab Documentation, 2004). With this software the following steps were performed per participant: 1) the T2-weighted image was loaded into the software and the signal intensity was normalized based on the histogram; 2) the previously described white matter ROI was loaded into the software and registered to the T2-weighted image using mutual information (Yoo and Lorensen, 2002) (Fig. 2.2); 3) the registered ROI was overlaid onto the T2-weighted image; 4) the registered ROI was manually adjusted to ensure the mask matches the NAWM – thus removing e.g. ICH and WMH from the mask – to avoid false positive and false negative PVS segmentations (Fig. 2.3); 5) a Frangi-vesselness filter (Frangi et al., 1998) was applied onto the T2-weighted images, within the ROI, and the filter threshold was manually adjusted to prevent both underestimation and overestimation of the PVS segmentation (Fig. 2.4); 6) the volume of the segmented PVS was calculated in mm^3^ and was divided by the NAWM ROI volume to account for the amount of WM in which PVS were segmented, resulting in the outcome variable PVS volume fraction (PVS_vf_) (Fig. 2.5). Two initial assessors (MRS and SvR, with over 3 and 15 years of experience, respectively) defined the NAWM ROI adjustment and filter threshold adjustment approach by consensus. Subsequently, the ROIs were generated and the filter thresholds were set by one assessor (MRS). With difficulty to generate ROI or set the filter threshold, cases were discussed with a neuroradiologist with over 20 years of experience (MAAvW). A subset of the baseline data was scored twice (MRS) to assess intra-rater variability (Supplementary Fig. S1).

### 2.4 Additional MRI markers

CSO-EPVS visual rating scale scores, as previously described (Potter et al., 2015), were obtained for the baseline in order to compare this with PVS_vf_. To give an estimation of disease severity in the current sample, the CAA cSVD burden score – a composite score of CSO-EPVS, cortical superficial siderosis, WMH, and cerebral microbleeds – was calculated as previously described and is presented in Table 1, Table 2 (Charidimou et al., 2016, Schipper et al., 2023). The additional MRI markers were scored by SvD and EAK with both over 5 years of experience (van Dijk et al., 2022).

**Table 1:** Baseline characteristics.

	Symptomatic D-CAA (n = 28)	Pre-symptomatic D-CAA (n = 15)	Controls > 50 years (n = 10)	Controls ≤ 50 years (n = 17)
Mean age ± SD [y] (range)	57 ± 6.4 (45 – 71)	39 ± 12.1 (20 – 55)	59 ± 5.5 (52 – 67)	36 ± 8.0 (20 – 47)
Female sex (%)	12 (43)	12 (80)	4 (40)	14 (82)
Mean BMI ± SD [kg/m^2^] (range)^A^	25.5 ± 3.1 (20.3 – 33.0)	25.9 ± 4.3 (20.0 – 35.7)	28.5 ± 4.9 (23.7 – 38.1)	23.5 ± 2.7 (20.2 – 30.7)
Median CAA cSVD score (IQR)^B^	5 (3 – 6)	1 (0 – 3)	0 (0 – 1)	0 (0 – 0)
CAA cSVD score 0	0	6	6	15
CAA cSVD score 1	0	3	3	2
CAA cSVD score 2	0	1	1	0
CAA cSVD score 3	7	2	0	0
CAA cSVD score 4	2	2	0	0
CAA cSVD score 5	9	0	0	0
CAA cSVD score 6	7	1	0	0
Hypertension (%)	11 (39)	1 (7)	5 (50)	0 (0)
Median CSO-EPVS visual rating scale (IQR)^C^	5 (5 – 5)	4 (3 – 5)	3 (3 – 4)	2 (2 – 3)
0: no CSO-EPVS	0	0	0	0
1: ≤ 10 CSO-EPVS	0	3	1	10
2: 11–20 CSO-EPVS	0	3	5	5
3: 21–40 CSO-EPVS	3	3	4	2
4: > 40 CSO-EPVS	23	6	0	0
Mean PVS_vf_ ± SD (range)	0.045 ± 0.026 (0.007 – 0.122)	0.026 ± 0.025 (0.003 – 0.080)	0.015 ± 0.005 (0.009 – 0.023)	0.006 ± 0.003 (0.002 – 0.014)

*Abbreviations*. D-CAA; Dutch-type Cerebral Amyloid Angiopathy. BMI; Body Mass Index. CAA; Cerebral Amyloid Angiopathy. cSVD; cerebral Small Vessel Disease. CSO-EPVS; enlarged perivascular spaces in the centrum semiovale. PVS_vf_: perivascular space volume fraction.

*A*. Three missing values in the symptomatic D-CAA, two missing values in pre-symptomatic D-CAA, and three missing values for controls ≤ 50 years for BMI.

*B*. Three missing values in the symptomatic D-CAA for CAA cSVD score.

*C*. Two missing values in the symptomatic D-CAA for CSO-EPVS visual rating scale.

**Table 2:** Follow-up characteristics.

	Symptomatic D-CAA (n = 16)	Pre-symptomatic D-CAA (n = 13)	Controls > 50 years (n = 3)	Controls ≤ 50 years (n = 8)
Available follow-up (%)	57	87	30	47
Median follow-up time [months] (IQR)	36 (35 – 41)	45 (35 – 51)	50 (50 – 55)	48 (37 – 52)
Mean age at baseline ± SD [y] (range)	57 ± 6.6 (47 – 71)	40 ± 12.0 (20 – 55)	62 ± 5.5 (56 – 67)	34 ± 9.0 (22 – 47)
Female sex (%)	9 (56)	10 (77)	2 (67)	7 (88)
Mean BMI at baseline ± SD [kg/m^2^] (range)^A^	24.6 ± 2.2 (20.3 – 29.4)	25.6 ± 4.6 (20.0 – 35.7)	26.4 ± 4.0 (23.7 – 31.0)	23.0 ± 1.7 (20.2 – 24.7)
Median CAA cSVD score at baseline (IQR)^B^	5 (3 – 6)	1 (0 – 3)	1 (0.5 – 1)	0 (0 – 0)
CAA cSVD score 0	0	5	1	7
CAA cSVD score 1	0	3	2	1
CAA cSVD score 2	0	0	0	0
CAA cSVD score 3	5	2	0	0
CAA cSVD score 4	2	2	0	0
CAA cSVD score 5	6	0	0	0
CAA cSVD score 6	2	1	0	0
Hypertension at baseline (%)	6 (37)	1 (8)	0 (0)	0 (0)
Mean baseline PVS_vf_ ± SD (range)	0.044 ± 0.030 (0.007 – 0.122)	0.028 ± 0.026 (0.003 – 0.080)	0.017 ± 0.006 (0.011 – 0.023)	0.006 ± 0.004 (0.002 – 0.014)
Mean follow-up PVS_vf_ ± SD (range)	0.045 ± 0.030 (0.009 – 0.116)	0.034 ± 0.029 (0.004 – 0.084)	0.021 ± 0.011 (0.011 – 0.034)	0.008 ± 0.007 (0.002 – 0.022)

*Abbreviations*. D-CAA; Dutch-type Cerebral Amyloid Angiopathy. BMI; Body Mass Index. CAA; Cerebral Amyloid Angiopathy. cSVD; cerebral Small Vessel Disease. PVS_vf_: perivascular space volume fraction.

*A*. Three missing values in the symptomatic D-CAA, two missing values in pre-symptomatic D-CAA, and two missing values for controls ≤ 50 years for BMI.

*B*. One missing value in the symptomatic D-CAA for CAA cSVD score.

### 2.5 Statistical analysis

Normality was assessed through histograms and Shapiro-Wilk tests.

To assess the relationship between age, mutation status, and PVS_vf_, we performed a multiple linear regression with PVS_vf_ as dependent variable and age and mutation status as independent variables.

To test the baseline group differences in PVS_vf_ – between symptomatic D-CAA mutation carriers, pre-symptomatic D-CAA mutation carriers, controls > 50 years, and controls ≤ 50 years – we performed Welch’s ANOVA with PVS_vf_ as dependent variable and group as independent variable. Subsequently, the Games-Howell test – including a correction for multiple testing – was performed to test mean differences between each pair.

The correlation between baseline PVS_vf_ and PVS visual rating scale score was assessed using Spearman’s rank correlation coefficient.

To assess group differences in longitudinal PVS_vf_ change, we performed Welch’s ANOVA with delta PVS_vf_ as dependent variable and group as independent variable. For this analysis the controls > 50 years and controls ≤ 50 years were grouped together due to small sample sizes. A comparison of the characteristics of participants that had a follow-up visit to participants that did not have a follow-up visit can be found in Supplementary Table S2.

To assess the intra-rater variability, we visualized the baseline scores of two ratings performed at different timepoints using a Bland-Altman plot (Supplementary Fig. S1) and calculated the intraclass correlation coefficient (ICC).

In addition, we performed a post-hoc linear regression per group to assess the association between baseline PVS_vf_ and the visually chosen filter threshold.

Lastly, post-hoc power analyses were performed, using ‘wanova_pwr.test’ for the Welch’s ANOVA testing baseline differences and for the Welch’s ANOVA testing longitudinal PVS_vf_ change.

## 3 Results

**Fig. 1:**
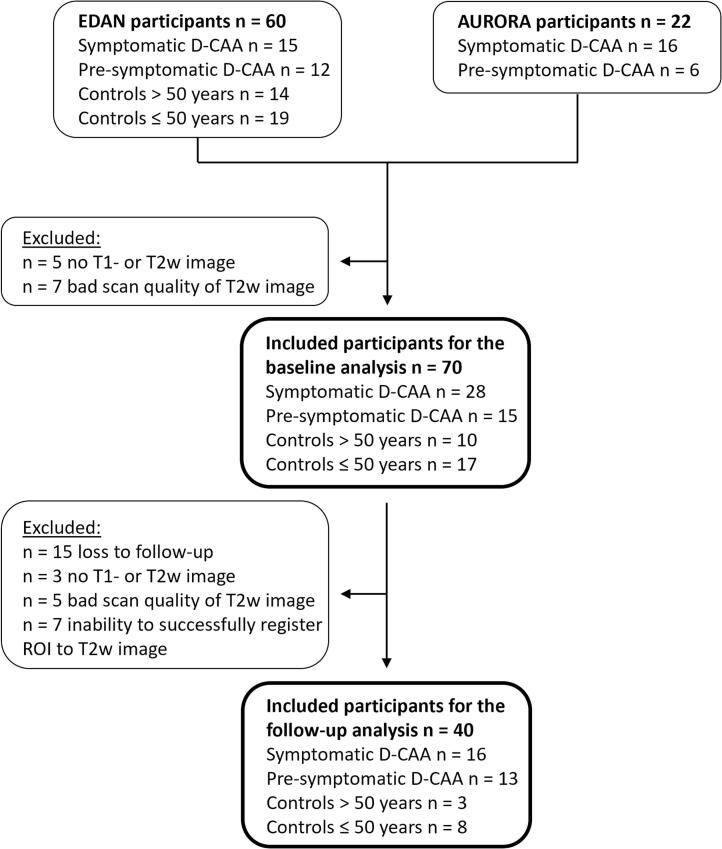
Inclusion flowchart. *Abbreviations.* D-CAA; Dutch-type Cerebral Amyloid Angiopathy. ROI; region of interest.

Detailed information on the included participants can be found in Fig. 1, Table 1, and Table 2. Normality of PVS_vf_ for the baseline analysis was observed in symptomatic D-CAA mutation carriers and controls > 50 years, and for the baseline data of the follow-up analysis in symptomatic D-CAA mutation carriers, controls > 50 years, and controls ≤ 50 years and for the follow-up data of the follow-up analysis in symptomatic D-CAA mutation carriers and controls > 50 years.

**Fig. 2:**
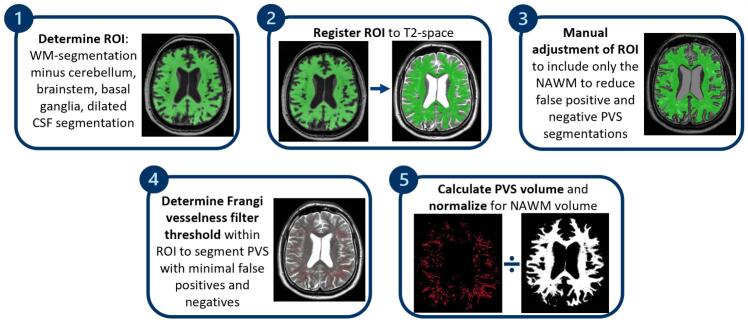
Perivascular space volume fraction analysis pipeline. The parameters for the Frangi-vesselness filter (used in step 4) were set to 10 scales ranging from sigma 1 to 3.5 (voxels), to accurately segment both slight and more pronounced dilatation of perivascular spaces. The filter threshold was subsequently determined to further minimize false positive and false negative perivascular space segmentations. *Abbreviations.* ROI; region of interest. WM; white matter. CSF; cerebrospinal fluid. NAWM; normal-appearing white matter. PVS; perivascular spaces.

**Fig. 3:**
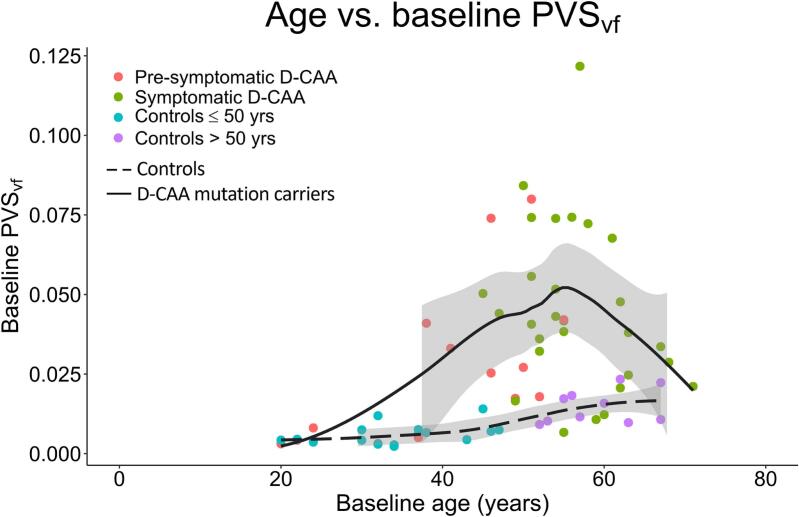
Scatterplot of age and perivascular space volume fraction at baseline, with locally estimated scatterplot smoothing regression lines including standard error for controls and D-CAA mutation carriers. *Abbreviations.* PVS_vf_; perivascular space volume fraction. D-CAA; Dutch-type Cerebral Amyloid Angiopathy.

Multiple linear regression proved age (B = 0.001, 95% CI [0.001, 0.001], *p* = 0.004) and mutation status (B = 0.025, 95% CI [0.015,0.036], *p* < 0.001, Adjusted R^2^ = 0.37) as predictors for PVS_vf_ (Fig. 3).

**Fig. 4:**
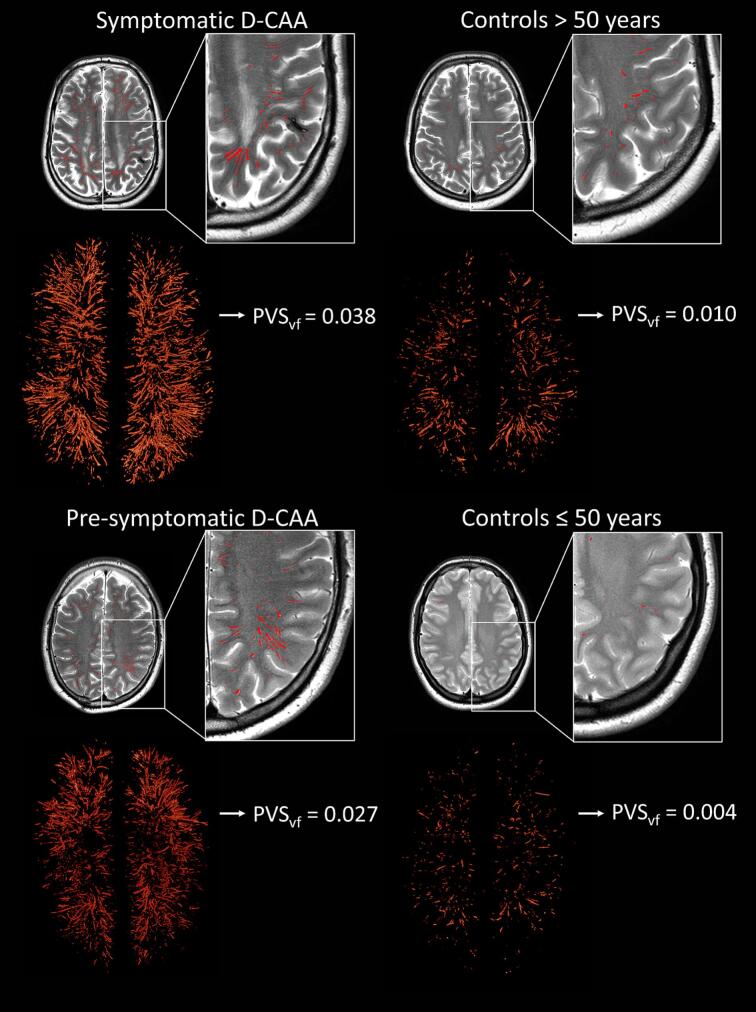
Example of perivascular space segmentations per group. The figure shows single slice examples of the perivascular space segmentations and volume rendered segmentations to illustrate whole-brain segmentations. *Abbreviations.* D-CAA; Dutch-type Cerebral Amyloid Angiopathy. PVS_vf_; perivascular space volume fraction.

**Fig. 5:**
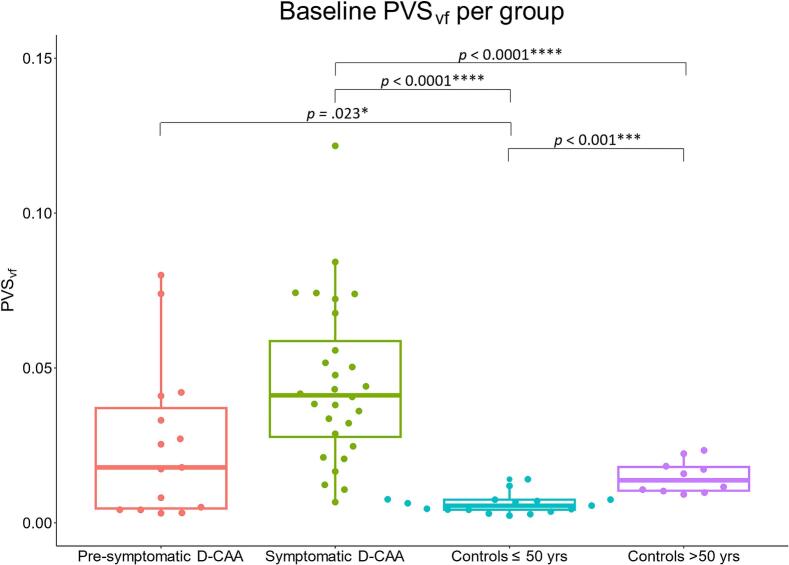
Boxplot overlaid with a beeswarm plot illustrating the distribution of perivascular space volume fractions per group. * = *p* ≤ 0.05, *** = *p* ≤ 0.001, **** = *p* ≤ 0.0001. *Abbreviations.* PVS_vf_; perivascular space volume fraction. D-CAA; Dutch-type Cerebral Amyloid Angiopathy.

Group differences were found in PVS_vf_ at baseline (F(3, 27) = 27.662, *p* < 0.001). Pairwise comparisons revealed a higher PVS_vf_ in symptomatic D-CAA mutation carriers compared to both control groups, in pre-symptomatic D-CAA mutation carriers compared to controls ≤ 50 years, and in controls > 50 years compared to controls ≤ 50 years (Table 1, Table 3, Fig. 4, Fig. 5). The effect size ω^2^ is 0.48 (large), indicating that 48% of the variance in PVS_vf_ was explained by group differences. Cohen’s d for pairwise effect sizes range from medium to large and are reported in Table 3.

**Table 3:** Games-Howell test statistics for baseline comparisons of perivascular space volume fraction per group.

Group comparison	Estimate	95% CI	Adjusted *p*-value	Cohen’s d
Pre-symptomatic vs. symptomatic D-CAA	0.019	−0.001, 0.039	0.063	0.76
Pre-symptomatic D-CAA vs. controls ≤ 50 years	−0.019	−0.035, −0.002	0.023*	1.15
Pre-symptomatic D-CAA vs. controls > 50 years	−0.010	−0.027, 0.007	0.378	0.55
Symptomatic D-CAA vs. controls ≤ 50 years	−0.038	−0.051, −0.025	< 0.0001****	2.62
Symptomatic D-CAA vs. controls > 50 years	−0.029	−0.042, −0.016	< 0.0001****	1.33
Controls ≤ 50 years vs. controls > 50 years	0.009	0.004, 0.014	< 0.001***	2.16

* = *p* ≤ 0.05, *** = *p* ≤ 0.001, **** = *p* ≤ 0.0001.

*Abbreviations*. D-CAA; Dutch-type Cerebral Amyloid Angiopathy.

**Fig. 6:**
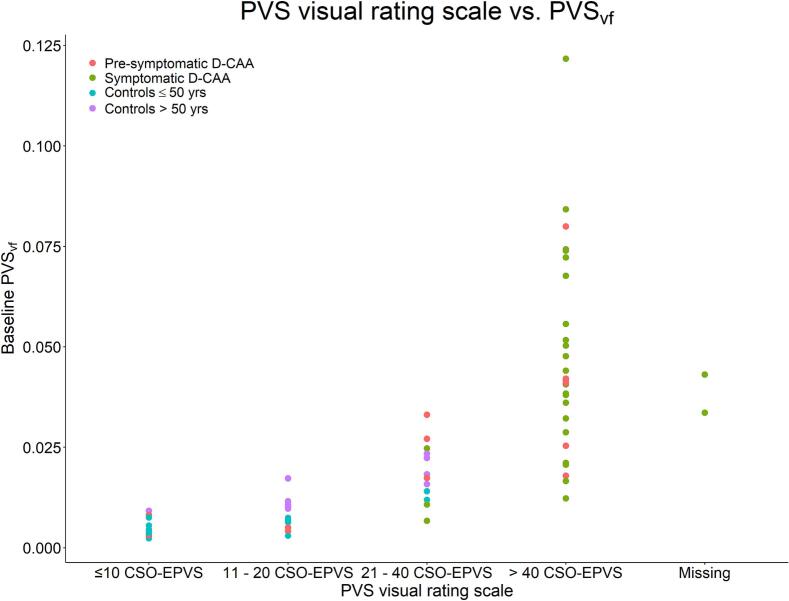
Scatterplot of baseline PVS_vf_ as function of the perivascular space visual rating scale per group. *Abbreviations.* PVS_vf_; perivascular space volume fraction. D-CAA; Dutch-type Cerebral Amyloid Angiopathy. CSO-EPVS; enlarged perivascular spaces in the centrum semiovale.

Spearman’s rank correlation coefficient of PVS_vf_ versus PVS visual rating scale score is 0.88 and thus shows strong and significant correlation (*p* < 0.001). PVS_vf_ was visualized as function of PVS visual rating scale score in Fig. 6.

**Fig. 7:**
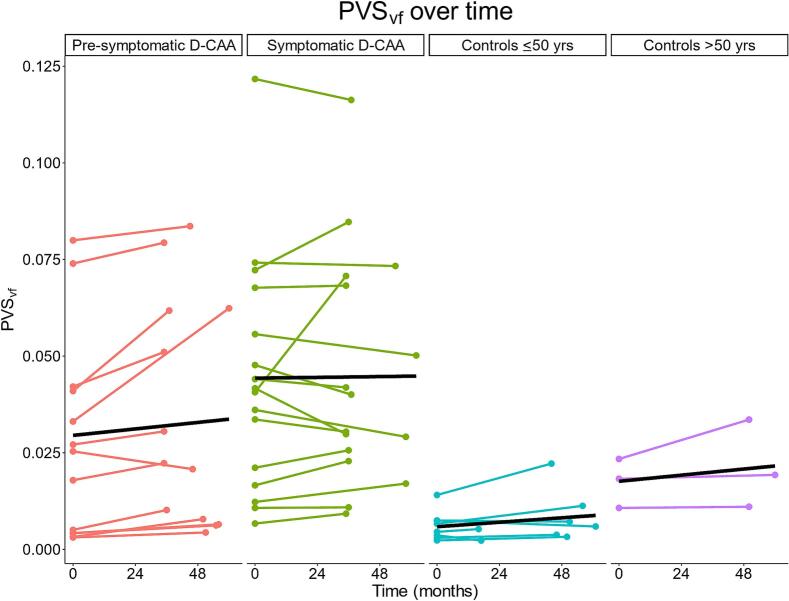
Scatterplot showing individual (colored lines) and group-level (black lines) follow-up changes in perivascular space volume fraction. Maximum follow-up time is 62 months. *Abbreviations.* PVS_vf_; perivascular space volume fraction. D-CAA; Dutch-type Cerebral Amyloid Angiopathy.

Sixty-five and a half percent of the D-CAA mutation carriers (81.3 percent of symptomatic D-CAA mutation carriers) fell within the highest visual rating category at baseline. No group differences were found in PVS_vf_ changes over time (F(3, 9) = 1.183, *p* = 0.369; Table 2, Fig. 7). The effect size ω^2^ is 0.02 (small), indicating that 2% of the variance in PVS_vf_ change was explained by group differences. Supplementary Table S2 shows that the participants with a follow-up visit are very similar to the participants who do not have a follow-up visit. Supplementary Fig. S2 shows follow-up NAWM ROI volume as a function of baseline NAWM ROI volume per group, showing that in most participants the NAWM-mask became smaller proving the importance to correct for ROI volume.

The intra-rater variability was visually inspected with a Bland-Altman plot (Supplementary Fig. S1) and showed stability in the two ratings. Stability of the ratings was especially observed with lower PVS_vf_. The ICC was 0.988, which indicates excellent intra-rater variability.

Post-hoc analysis showed a positive linear association between baseline PVS_vf_ and filter threshold in pre-symptomatic D-CAA mutation carriers (B = 3.731, 95% CI [0.360, 7.102], *p* = 0.033, Adjusted R^2^ = 0.25). Non-significant associations were found between baseline PVS_vf_ and filter threshold in symptomatic D-CAA mutation carriers, controls > 50 years, and controls ≤ 50 years (Supplementary Table S3 and S4, Supplementary Fig. S3).

Post-hoc power analyses of the Welch’s ANOVA tests for baseline group differences in PVS_vf_ and longitudinal group differences in PVS_vf_ change, showed that the power was 0.93 and 0.90 at an alpha level of 0.05, respectively. These power calculations suggest that the tests were well-powered to detect significant differences between groups.

## 4 Discussion

The results of this study are four-fold. First, we found a higher PVS_vf_ in symptomatic D-CAA mutation carriers compared to controls ≤ 50 and > 50 years, in pre-symptomatic D-CAA mutation carriers compared to controls ≤ 50 years, and in controls > 50 years compared to controls ≤ 50 years. Second, we found no group differences in PVS_vf_ change after three to five years. Third, the intra-rater variability showed excellent reliability of the rating, stability of the ratings was most pronounced for lower PVS_vf_ (Supplementary Fig. S1). Last, we found a positive relation between baseline PVS_vf_ and chosen Frangi-vesselness filter threshold in pre-symptomatic D-CAA mutation carriers.

Increased PVS_vf_ in pre-symptomatic D-CAA mutation carriers compared to similar-age controls is a novel finding and is in line with the recently introduced pathophysiological framework on the CAA disease progression, which suggests that non-hemorrhagic injury occurs early on in CAA^4^. Increased PVS_vf_ in pre-symptomatic D-CAA mutation carriers may represent the elevated sensitivity of the current volumetric, whole-brain PVS assessment – stressing the relevance of volumetric measurements. MRI-markers that have been established in the early stage of CAA are WMHs, cortical microinfarcts, and reduced vasoreactivity (Koemans et al., 2023, van Rooden et al., 2016, van Opstal et al., 2017). With an automated pipeline, PVS quantification might be an asset to clinical assessment, in contrast to measurements of cortical microinfarcts and vasoreactivity – due to difficulty in assessment – especially since T2-weighted sequences are often part of neuroimaging protocols. Assessment of enlarged PVS in early stages of CAA may contribute to an early diagnosis, however, a strictly lobar hemorrhagic lesion is also required for the diagnosis – as enlarged PVS are not specific for CAA (Charidimou et al., 2022, Duering et al., 2023). A higher PVS_vf_ was also found in symptomatic D-CAA mutation carriers in comparison to similar-age controls. This is in line with previous PVS assessments based on visual rating scales and single slice quantification (Martinez-Ramirez et al., 2018, Charidimou et al., 2017). Fig. 6 shows overlap in PVS_vf_ between different categories of the visual rating scale, which could indicate that visual rating scale scores may not be representative of whole white matter PVS. In addition, the highest category of the visual rating scale encompasses a large range of PVS_vf_, stressing the ceiling effect that is present in the visual rating scale.

Over time, we found no group differences in PVS_vf_ change, as was also not shown previously by means of CSO-EPVS visual rating scale (van Dijk et al., 2022). However, in three cases – one symptomatic and two pre-symptomatic D-CAA mutation carriers – a large increase (greater than 2 SD) in PVS_vf_ was detected. These participants already fell within the highest category of the visual rating scale at baseline (Fig. 6, Fig. 7). In some cases, especially in symptomatic D-CAA mutation carriers, reductions in PVS_vf_ can be detected. These reductions may be the result of NAWM ROI shrinkage due to new ICH and WMH that will occur especially in CAA compromised areas. Therefore, there might be an underestimation of the rate of growth. This is also illustrated through lower PVS_vf_ in older symptomatic D-CAA and the inverted U-shape relation between age and PVS_vf_ in D-CAA (Fig. 3, Fig. 7).

Since Frangi-vesselness filter threshold affects the segmented PVS volume, one might expect a bias towards higher volumes with lower filter thresholds. However, the current results indicate no bias towards higher PVS volumes with lower filter thresholds but rather the opposite. The higher filter threshold with higher PVS_vf_ may result from increased noise levels and heterogenous signal intensities as observed throughout the brain, potentially due to increased restlessness while scanning or (micro)structural white matter variations in more affected participants. The fact that this relation is only seen in pre-symptomatic D-CAA is likely because of the heterogeneity in this group that is reflected by e.g. the wide age and CAA sCVD score range (Table 1).

The current study has several limitations. First, successful PVS_vf_ analysis is reliant on good image quality, and thus, poor image quality is a reason for exclusion in this study (Fig. 1). High noise levels and image artefacts limit accurate PVS assessment and segmentation. Although this limitation generally affects the assessment of MRI markers, PVS assessment may be affected more profoundly by image degradation as PVS are typically tiny structures. Second, exclusions in this study based on loss to follow-up, bad image quality, and the inability to perform accurate registrations may indicate that the current baseline and follow-up sample reflects more healthy participants, especially biasing the symptomatic D-CAA mutation carriers and older controls. However, our comparison of participants with and without follow-up visit, did not show any substantial differences. Third, the current method is time-consuming and labor-intensive. Adjustments of the preliminary ROI and assessment of the filter threshold took 90 min on average and could take up to 180 min per scan. This stresses the need for advances in PVS assessment, e.g. through adoption of machine learning (Lan et al., 2023, Huang et al., 2024, Rashid et al., 2023) – as is currently increasingly used in automatization of segmentations – especially if this measure is to be included in clinical practice. However, because such methods are currently not trained on CAA-data and are therefore expected to perform inadequate in the presence of profound pathology, such as WMH and ICHs, we opted for a semi-automatic approach. Moreover, we decided not to apply existing deep-learning-based and other vesselness filter-based pipelines to compare our current method with, as we assume this will not provide a fair comparison. Fourth, it is important to take acquisition resolution and imaging field strength into consideration when interpreting PVS volume results and especially when comparing these results with future studies. Higher acquisition resolutions and stronger imaging field strengths may increase sensitivity to smaller PVS and vice versa. For the current study very similar scan sequences were used in the two cohorts, of which we assume no differences in segmentations. All of the follow-up scans within participants were performed with the same scan settings, eliminating a potential effect of scan sequence on follow-up differences. Although the analyzed T2-weighted images had a sub-millimeter in-plane acquisition resolution of 0.50 mm by 0.56 mm, we were limited to the slice thickness of 3.0 – 3.6 mm that led to partial volume effects and reduced sensitivity in the feet-head direction. Finally, the current sample size restricted the statical power of our analyses and our ability to adjust for confounders (Barisano et al., 2021). Although we have a small sample size, as D-CAA is a rare disease, we used the largest cohort of D-CAA currently available.

The main strength of the current study is that we used a novel method to quantify relative whole-brain PVS volume in pre-symptomatic and symptomatic D-CAA mutation carriers. Where former visual rating scales are limited to a single slice, arbitrary scales with a strong ceiling effect, and PVS count, the current whole-brain semi-automatic PVS quantification enables sensitivity without ceiling effects. In addition, the effect of reduced sensitivity to PVS in the through-plane direction when MRI is acquired with anisotropic voxels, will be reduced when considering whole-brain PVS. Furthermore, the semi-automatic PVS_vf_ assessment allowed for a personalized approach to ensure that the ROI fits the NAWM and to set filter thresholds per participant. This is an important part of the current method as using the raw WM segmentation as ROI and an uniform filter threshold would lead to many false positive and negative PVS segmentations (Supplementary Fig. S4). However, a personalized approach may be more subjective to bias. We limited bias by randomizing participants, subgroups, and timepoints in the PVS analysis. In addition, higher filter thresholds were applied with higher PVS_vf_ in pre-symptomatic D-CAA mutation carriers, which is more likely explained by increased noise levels and white matter variations in more affected patients than by subjective bias. In addition, the ICC showed excellent reliability between ratings done at different timepoints, indicating within rater robustness of the measure. Also, the current study population – D-CAA mutation carriers – allowed us to study PVS already in the pre-symptomatic phase of the disease, enabling early detection of increased PVS_vf._ Finally, inclusion of similar-age controls allowed us to distinguish PVS_vf_ (progression) in D-CAA mutation carriers from normal aging.

To conclude, increased PVS_vf_ in pre-symptomatic D-CAA mutation carriers may introduce the potential of PVS_vf_ as early marker. We found no group differences in PVS_vf_ changes over three to five years follow-up. Intra-rater observations indicate that the current methodology appears quite robust even in the current study population that included subjects with rather severe brain pathology. Taken altogether, the current method is a promising measure for the assessment of PVS_vf_. Future technological developments can further improve this method by increasing scan resolution, for example by moving to ultra-high field MRI or 3D acquisition schemes, and by reducing processing time. PVS segmentation would benefit from an automated pipeline designed specifically for CAA in order to take into account CAA-related pathology that will likely disrupt existing pipelines and neural networks that have been trained on non-CAA data. Segmentations from the current study could function as training data for such a neural network.

## CRediT authorship contribution statement

**Manon R. Schipper:** Writing – review & editing, Writing – original draft, Visualization, Validation, Software, Resources, Project administration, Methodology, Investigation, Formal analysis, Data curation, Conceptualization. **Thijs W. van Harten:** Writing – review & editing. **Arie-Tjerk Razoux-Schultz:** Writing – review & editing, Methodology. **Kanishk Kaushik:** Writing – review & editing, Data curation. **Lydiane Hirschler:** Writing – review & editing, Visualization. **Sabine Voigt:** Writing – review & editing, Data curation. **Ingeborg Rasing:** Data curation. **Emma A. Koemans:** Data curation. **Rosemarie van Dort:** Writing – review & editing. **Reinier G.J. van der Zwet:** Writing – review & editing. **Sanne E. Schriemer:** Writing – review & editing, Data curation. **Erik W. van Zwet:** Writing – review & editing. **Jeroen van der Grond:** Writing – review & editing. **Mark A. van Buchem:** Writing – review & editing. **Steven M. Greenberg:** Writing – review & editing, Funding acquisition. **Marieke J.H. Wermer:** Writing – review & editing, Supervision, Funding acquisition. **Matthias J.P. van Osch:** Writing – review & editing, Supervision, Methodology, Funding acquisition, Conceptualization. **Marianne A.A. van Walderveen:** Writing – review & editing, Supervision, Methodology. **Sanneke van Rooden:** Writing – review & editing.

## Declaration of Competing Interest

The authors declare that they have no known competing financial interests or personal relationships that could have appeared to influence the work reported in this paper.

## Data Availability

Data will be made available on request.
