# Supplementary material for: Cross-sectional and longitudinal quantification of total white matter perivascular space volume fraction in Dutch-type Cerebral Amyloid Angiopathy
Source: Neuroimage Clin. 2025 Apr 5;46:103778. doi: 10.1016/j.nicl.2025.103778 (PMC12013478; doi:10.1016/j.nicl.2025.103778)
Supplement: Supplementary Data 1 [file mmc1.docx]

**Supplementary Table 1.** 3.0 Tesla MRI protocol of the AURORA study including the relevant scans for the current study.

|  | 3D T1-weighted sequence | T2-weighted sequence | 3D FLAIR sequence | SWI sequence |
| --- | --- | --- | --- | --- |
| TR [ms] | 9.7 | 4744 | 4800 | 31 |
| TE [ms] | 4.6 | 80 | 280 | 7.2 |
| Flip angle [degrees] | 8 | 90 | TI: 1650 ms | 17 |
| Number of slices | 130 | 48 | 321 | 130 |
| Slice thickness [mm] | 1.20 | 3.00 | 0.60 | 1.00 |
| Interslice gap [mm] | 0 | 0 | 0 | 0 |
| FOV [mm] | 217x172x156 | 220x176x144 | 250x250x180 | 230x190x130 |
| Voxel size [mm] | 1.2x1.2x1.2 | 0.5x0.6x3.0 | 1.0x1.0x0.6 | 0.6x0.6x1.0 |
| Scan duration [min] | 2:48 | 2:13 | 4:43 | 3:31 |

*Abbreviations.* FLAIR; Fluid Attenuated Inversion Recovery. SWI; Susceptibility Weighted Imaging. TR; repetition time. TE; echo time. TI; inversion time. FOV; Field of View.

**Supplementary Table 2.** Mean and frequency values of characteristics and relevant markers of participants that are included in the follow-up analysis and participants who are not included in the follow-up. Both control groups, >50 and ≤ 50 years, are taken together as they are not subdivided in the follow-up analysis.

|  | Symptomatic D-CAA | | Pre-symptomatic D-CAA | | | | Controls | |
| --- | --- | --- | --- | --- | --- | --- | --- | --- |
|  | *Mean value  follow-up* | *Mean value  no follow-up* | | *Mean value  follow-up* | *Mean value  no follow-up* | *Mean value  follow-up* | | *Mean value  no follow-up* |
| Age at baseline | 57 | 56 | | 40 | 37 | 42 | | 45 |
| BMI at baseline | 25 | 27 | | 26 | 28 | 24 | | 26 |
| CAA cSVD score at baseline | 4.3 | 5.1 | | 1.8 | 1 | 0.3 | | 0.3 |
| CSO-EPVS visual rating scale at baseline | 4.9 | 5.0 | | 3.9 | 3.0 | 2.9 | | 2.8 |
| PVS_vf_ at baseline | 0.044 | 0.047 | | 0.028 | 0.013 | 0.009 | | 0.009 |
|  | *Frequency  follow-up* | *Frequency  no follow-up* | | *Frequency  follow-up* | *Frequency  no follow-up* | *Frequency  follow-up* | | *Frequency  no follow-up* |
| Female sex (%) | 9 (56%) | 3 (25%) | | 10 (89%) | 2 (100%) | 9 (82%) | | 9 (56%) |
| Hypertension at baseline (%) | 6 (38%) | 5 (42%) | | 1 (8%) | 0 (0%) | 0 (0%) | | 5 (31%) |

*Abbreviations.* D-CAA; Dutch-type Cerebral Amyloid Angiopathy. BMI; Body Mass Index. CAA; Cerebral Amyloid Angiopathy. cSVD; cerebral Small Vessel Disease. CSO-EPVS; enlarged perivascular spaces in the centrum semiovale. PVS_vf_: perivascular space volume fraction.

Scores for CSO-EPVS visual rating scale at baseline are: 1 = no CSO-EPVS, 2 = ≤10 CSO-EPVS, 3 = 11 – 20 CSO-EPVS, 4 = 21 – 40 CSO-EPVS, 5 = >40 CSO-EPVS.

**Supplementary Table 3.** Summary of baseline Frangi vesselness filter threshold per group.

|  | Symptomatic D-CAA  (n = 28) | Pre-symptomatic  D-CAA (n = 15) | Controls > 50  years (n = 10) | Controls ≤ 50  years (n = 17) |
| --- | --- | --- | --- | --- |
| Mean Frangi vesselness filter threshold ± SD (range) | 0.027 ± 0.003 (0.022 – 0.034) | 0.026 ± 0.004 (0.021 – 0.032) | 0.023 ± 0.002 (0.021 – 0.024) | 0.022 ± 0.002 (0.018 – 0.025) |

*Abbreviations.* D-CAA; Dutch-type Cerebral Amyloid Angiopathy.

**Supplementary Table 4.** Post-hoc linear regression statistics regarding perivascular space volume fraction in relation to the Frangi vesselness filter threshold per group.

|  | Unstandardized  B-value | df | t-statistic | 95% CI | *p-*value | Adjusted R^2^ |
| --- | --- | --- | --- | --- | --- | --- |
| Symptomatic D-CAA | 2.409 | 27 | 1.668 | -0.560, 5.378 | 0.107 | 0.062 |
| Pre-symptomatic D-CAA | 3.731 | 14 | 2.391 | 0.360, 7.102 | 0.033* | 0.252 |
| Controls > 50 years | 2.067 | 10 | 2.169 | -0.056, 4.190 | 0.055 | 0.212 |
| Controls ≤ 50 years | 0.773 | 13 | 1.883 | -0.114, 1.660 | 0.082 | 0.154 |

*Abbreviations.* D-CAA; Dutch-type Cerebral Amyloid Angiopathy. df; degrees of freedom.

* = *p* ≤ 0.05.

**Supplementary Figure 1**


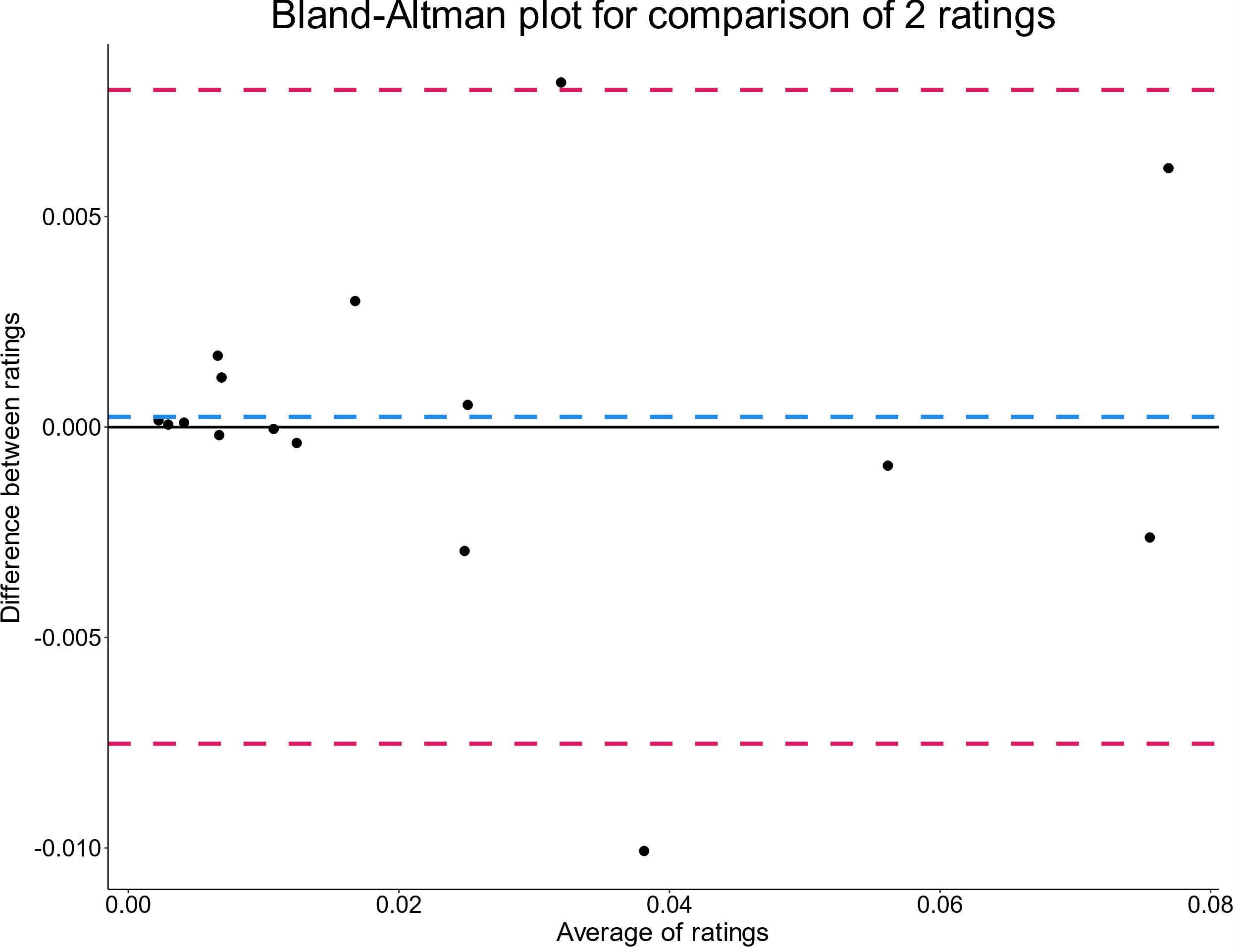
Bland-Altman plot of the intra-rater difference in perivascular space volume fraction.

**Supplementary Figure 2**


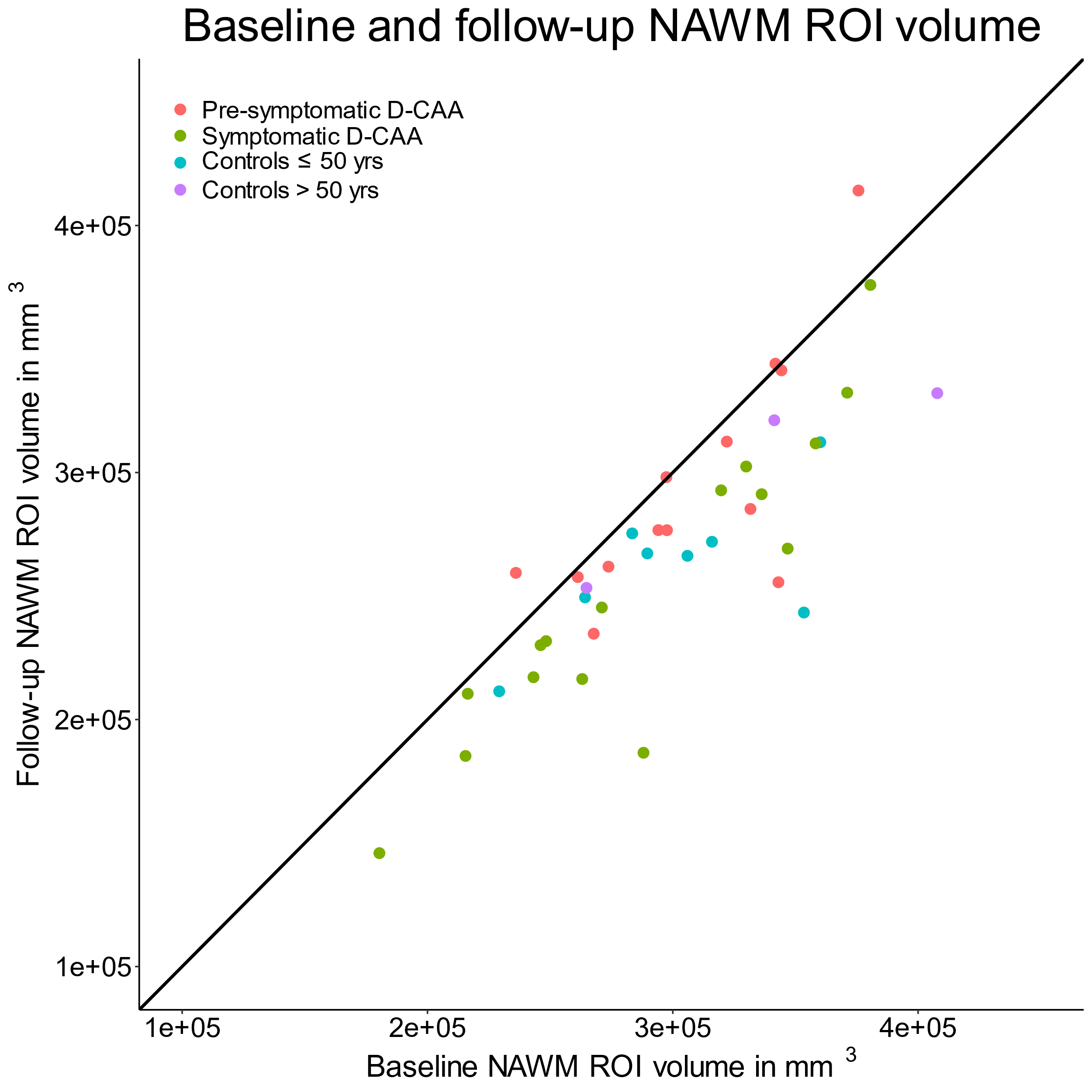


Scatterplot of baseline normal appearing white matter region of interest volume as compared to follow-up normal appearing white matter region of interest volumes.

*Abbreviations.* NAWM; normal appearing white matter. ROI; region of interest. D-CAA; Dutch-type Cerebral Amyloid Angiopathy.

**Supplementary Figure 3**

**
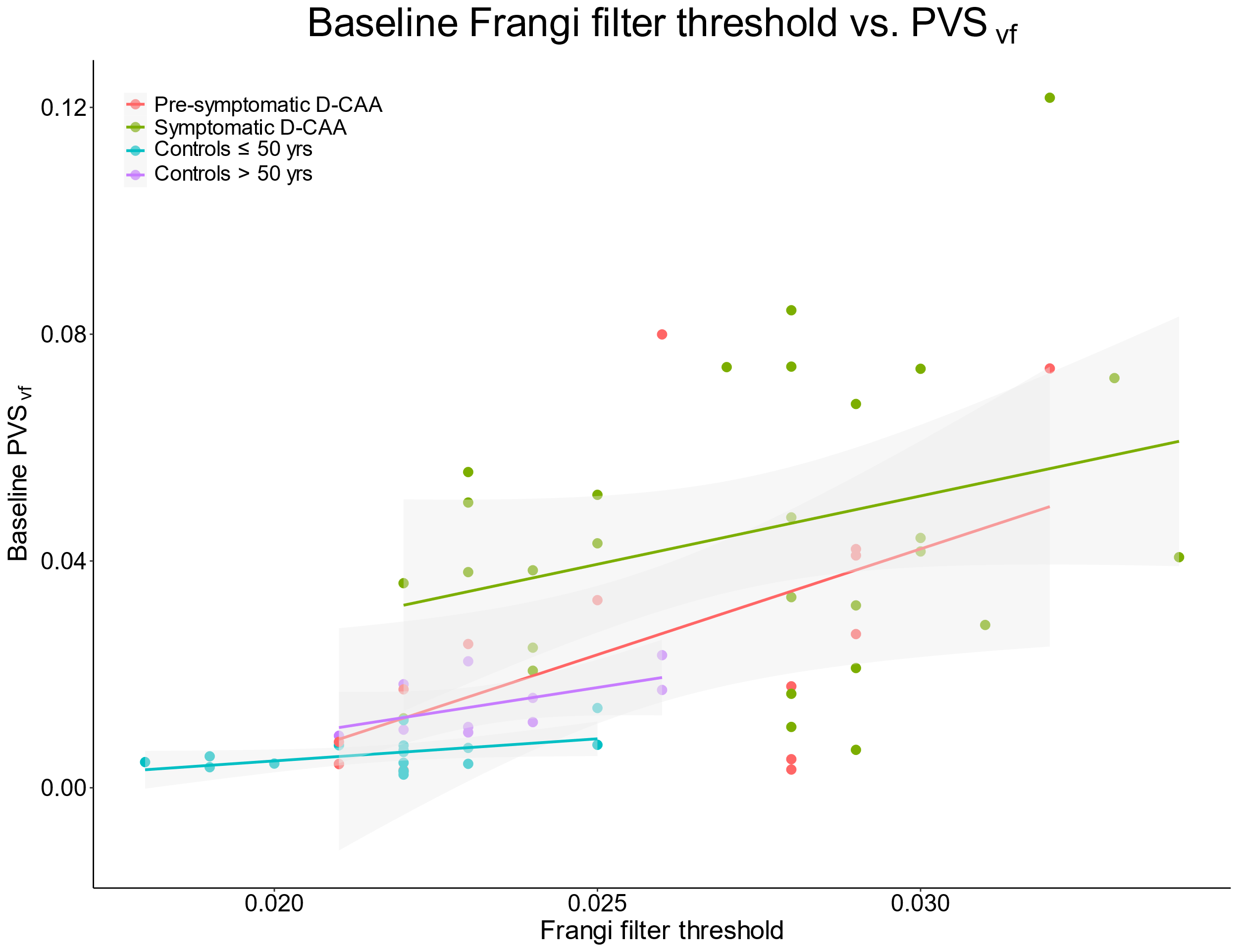
**

Scatterplot of baseline perivascular space volume fraction as function of Frangi vesselness filter threshold with regression lines and standard error ranges per group.

*Abbreviations.* PVS_vf_; perivascular space volume fraction. D-CAA; Dutch-type Cerebral Amyloid Angiopathy.

**Supplementary Figure 4**


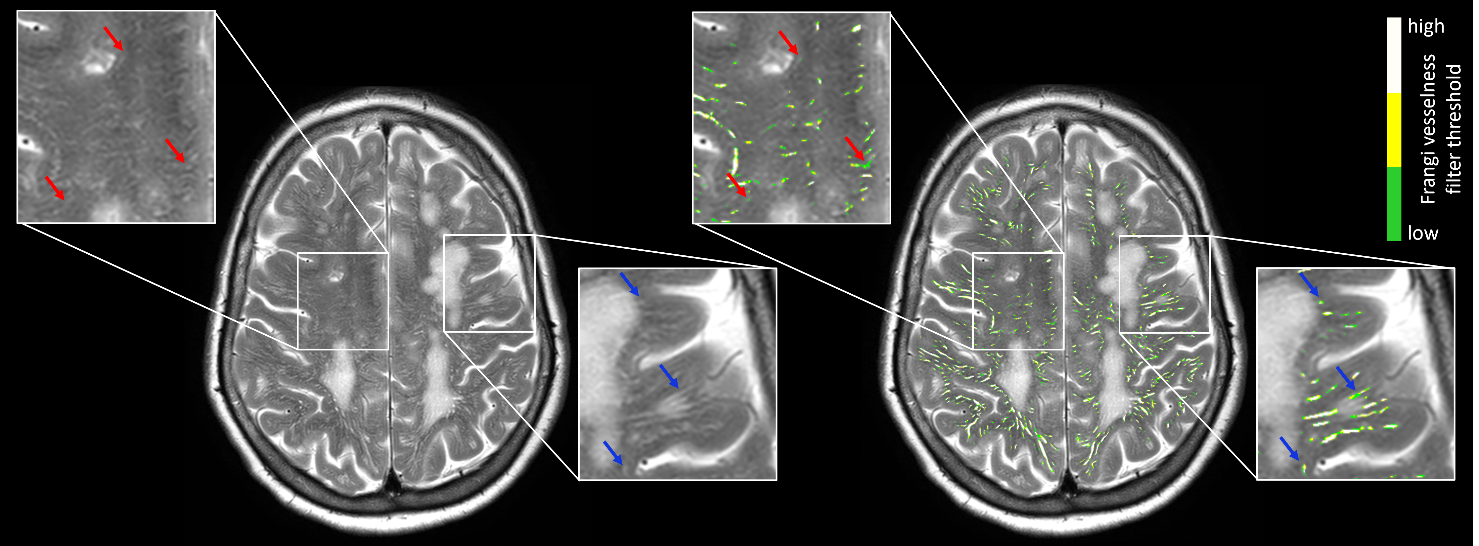
Illustration of perivascular space segmentations based on varying Frangi vesselness filter thresholds as additive overlays onto a T2-weighted image. The white overlay reflects the segmentation based on a high threshold, the overlay that also includes yellow reflects the segmentation based on a medium threshold, and the overlay that also includes green reflects the segmentation based on a low threshold – thresholds were 0.032, 0.029, and 0.026 respectively. For this participant, a threshold of 0.029 was used for the perivascular space analysis. This example illustrates that a lower threshold can result in the inclusion of more noise (indicated by the red arrows) while a higher threshold can result in the exclusion of perivascular spaces (indicated by the blue arrows).
